# The establishment and utility of Sweha-Reg: a Swedish population-based registry to understand hereditary angioedema

**DOI:** 10.1186/1471-5945-7-6

**Published:** 2007-11-30

**Authors:** Lotus Mallbris, Patrik Nordenfelt, Janne Björkander, Anders Lindfors, Sonja Werner, Carl-Fredrik Wahlgren

**Affiliations:** 1 Department of Medicine, Unit of Dermatology, Karolinska University Hospital Solna, Karolinska Institutet, Stockholm, Sweden; 2 Department of Clinical and Experimental Medicine (IKE), Faculty of Health Sciences, Linköping University, Linköping, Sweden; 3 Department of Clinical and Experimental Medicine, County Hospital Ryhov, Jönköping, Sweden; 4 Department of Pediatrics, Karolinska University Hospital Solna, Karolinska Institutet, Stockholm, Sweden; 5 Department of Respiratory Medicine and Allergology, University hospital, Lund, Sweden

## Abstract

**Background:**

The importance of acquiring comprehensive epidemiological and clinical data on hereditary angioedema has increasingly caught the attention of physicians and scientists around the world. The development of networks and creation of comprehensive policies to improve care of people suffering from rare diseases, such as hereditary angioedema, is a stated top priority of the European Union.

Hereditary angioedema is a rare disease, that it may be life-threatening. Although the exact prevalence is unknown, current estimates suggest that it is 1/10,000–1/150,000 individuals. The low prevalence requires combined efforts to gain accurate epidemiological data on the disease and so give us tools to reduce morbidity and mortality, and improve quality of life of sufferers.

**Methods:**

Sweha-Reg is a population-based registry of hereditary angioedema in Sweden with the objectives of providing epidemiological data, and so creates a framework for the study of this disease. The registry contains individual-based data on diagnoses, treatments and outcomes.

**Conclusion:**

The present manuscript seeks to raise awareness of the existence of Sweha-Reg to stimulate the international collaboration of registries. A synthesis of data from similar registries across several countries is required to approach an inclusive course understanding of HAE.

## Background

Hereditary angioedema (HAE) is a rare and potentially life-threatening condition that clinically manifests itself as acute attacks of facial, laryngeal, genital and peripheral swelling and/or recurrent abdominal pain, with a significant effect on the quality of life of sufferers [1]. The condition can have profound physical and psychological effects, which can prevent a normal social activity.

The disease results from deficient production (HAE-I) or function (HAE-II) of C1 esterase inhibitor (C1-INH), and is caused by mutation of the *C1NH *gene on chromosome 11 [2]. To date, approximately 190 different *C1NH *mutations are known [3]. The disorder, which has an dominant hereditability, has traditionally been described in 2 types: HAE-I and HAE-II [4,5]. However, recently it has been proposed a third type (HAE-III), with normal C1-INH activity and unknown pathomechanism [6,7]. Although recent reports proposed increased activity of coagulation factor XII (Hageman factor) as a possible cause of HAE type III [8,9].

Despite the substantial immunogenetic knowledge that has been amassed, different aspects of HAE are only partially understood. In Sweden, basic epidemiological parameters such as prevalence and incidence are lacking, and patient materials investigated are very small and often only include selected subgroups. Moreover, there is no national consensus on what constitutes state-of-the-art evidence-based treatment and consequently patients are treated differently at various centers. Also, lack of comprehensive epidemiological data has further decreased the value of case reports that appear to link purported eliciting factors to the occurrence of attacks. Accordingly, a team of physicians and scientists in Sweden have initiated a national population-based patient register (Sweha-Reg) with the objective of gaining a better understanding of HAE, including its epidemiology, clinic and management. The present manuscript presents the Sweha-Reg and is also an effort to stimulate the international collaboration of registries. Cross linking of similar registries across several countries is required to approach an inclusive presentation and course understanding of HAE.

## Methods

Historically, Swedish health care has generally been population-based, since it is mainly funded by taxes and private health-care providers have been scarce.

Since January 1, 1947, all inhabitants of Sweden have been assigned a 10-digit national registration number, which is a unique personal identifier. Since it is used in most registries it provides an opportunity to unambiguously link information from different sources.

### Sweha-Reg

Sweha-Reg is a population-based census of HAE in Sweden, established to achieve a baseline epidemiological description, and to provide a framework to study quality of life, and to further characterize the disease. The registry attempts to capture data on all individuals with diagnosed or suspected HAE type I and II in Sweden by using multiple recruitment channels, and to record enough clinical information, including biochemical diagnostic markers to assure a case definition with high sensitivity and specificity, accurately excluding those with non-hereditary angioedema. Sweha-Reg is a nation-wide registry that serves the entirety of Sweden, which is estimated to have a population of 9.1 million people.

Cases are prospectively followed over time for disease course, efficacy, safety and outcome of different treatments, for co-morbidities and quality of life. Furthermore, we have used the Swedish Population Registry to identify a set of control subjects matched to the first 100 adult cases (> 18 yrs old) enrolled during early 2007. The controls are selected from the Stockholm County as a stratified random sample, taking age and sex into consideration. Three control subjects are selected per adult case.

Additionally, biological specimens, including serum, plasma and whole-blood are collected for studying the biochemical, immunological and genetical background of the patients. Data obtained from this biobank (Sweha-Bank) can be useful for describing common polymorphisms in Sweden, establishing a basis for biobank-based post-genome studies, integrating phenotype and genotype data. The stored biological specimens are useful also for other pertinent research.

### Case definition and recruitment

In brief, the cases comprise individuals at any age with a diagnosis and/or treatment of HAE type I and II, and who live in Sweden. Diagnosis and classification of HAE is made using established criteria and terminology [10]. Only subjects with a convincing clinically and laboratory diagnosis of HAE are included. In situations in which the diagnosis is uncertain, additional previous medical records are requested to determine an accurate diagnosis.

Patients are recruited nationwide. In order to enroll as many cases as possible, multiple recruitment sources are used, such as physicians and clinics specialized in HAE, hospital discharge databases and periodic correspondence with all medical directors of dermatologic, pediatric, allergologic and internal medicine clinics at Swedish hospitals, as well as the immunologic HAE-laboratories in Lund and Uppsala. Additional cases are recruited through advertisements, campaigns on the website and magazine of the Primary Immunodeficiency Organization (PIO) in Sweden.

Once a potential case is identified, informed consent to approach the patient to join the registry and to release medical information is obtained via the treating physician. This process is most straightforward for patients treated by the registry investigators. To enlist cases handled by other physicians, the registry sends the survey information to the treating physician to pass on to patients. Thus, all patients are contacted through their treating physicians. Unlike some other population-based registries, such as The Swedish Cancer Registry, there is no requirement on the part of hospitals or treating physicians to report HAE cases. Moreover, the Sweha-Reg requires consent for patient participation. These issues add to the challenge of completeness of case-participation. In order to optimize participation, there is no absolute requirement for patients to provide blood samples. In addition self referral is possible through contacting the survey's telephone hotline or e-mail service. By means of these services patients and physicians are also able to receive advice and counsel with regard to any question related to the Sweha-project and/or HAE in general.

Moreover, close collaboration within the Sweha study group, involving HAE-leading physicians in Sweden, plays a crucial role in the recruitment process. This study group also disseminates information on Sweha-Reg and fosters a constructive collaboration between investigators and treating physicians.

### Participation

Participation involves answering a mailed questionnaire, followed by attending a standardized telephone interview by a physician, and for those who live near one of the survey's qualified laboratory centers, to provide blood samples for laboratory analyses and for storage in Sweha-Bank.

Written informed consent is obtained from the study participants or their parents (for individuals aged < 18 yrs) prior to the telephone interview. Parental interviews are conducted for patients with an age less than 12 yrs of age. Control individuals answer an identical questionnaire as cases, but do not attend the telephone interview.

Blood samples, a total amount of approximately 40 ml (less in young children), are collected at one of the qualified laboratories, assuring the appropriate systems for biological specimen collection, processing and storage. The labs are located in 4 different areas in Sweden (Lund, Jönköping, Göteborg and Stockholm). The Sweha-survey, including the registry and biobank, is approved by the ethics committee of the Karolinska Institutet, Sweden. The survey is performed according to the Declaration of Helsinki Principles.

### Questionnaire

Registry participants are mailed a questionnaire with up to 3 reminder follow-up letters if it is not returned. For patient's convenience the questionnaire is in paper format. However Sweha-Reg, consisting of data from the questionnaire and the telephone interview, exists in digital format which could easily be adapted as a web-based register, if such a design is found beneficial. Once the questionnaire is returned it is transferred to the digital format.

Information on demographic data, social, educational, economical, and health status, quality of life, family history of HAE, co-morbidities, potentially predisposing trigger factors surrounding onset and new attacks, previous and ongoing medication and attacks characterization and severity of symptoms are obtained by answering 38 questions.

### Telephone interview

The telephone interview is structured to capture the natural course of HAE and the efficacy, safety and outcome of different treatments in patients and the extent of usage of health and social services. Furthermore, information about potential precipitating factors and co-morbidities, provided through the mailed questionnaire, is verified and missing or unclear data are completed. Additional information includes family history of HAE. Besides, the telephone interview provides patients with the opportunity to raise questions related to their disease and its treatment.

### Confidentiality of Patient Records

Due to patient confidentiality requirements, all subjects receive a unique study-identification code, which anonymizes the records. Only the registry's main investigators (CFW and LM) know the code and are able to link an individual report to an individual patient. Access to data will initially be restricted to the Sweha study group physicians participating in the concerted action but will eventually be open, on request, to any HAE-treating physician. Also, clinicians contributing to the register will have access to the data of their own patients, and will obtain (by request) feedback such as individual or group reports.

The survey board, consisting of the authors of the present manuscript, manages the anonymized database and may access aggregated reports but not individual data sets. No single member of the board can use or publish the data without the consent of the board and the contributors as a whole.

### Cross-linking of registries

One of the Sweha-Reg's objectives is to be able to share and compare data with other similar HAE-registries in other nations. In this context, some of the questions are designed to be compatible with data in the European HAE registry [11]. Moreover, by using the unique Swedish personal identification number, we are able to crosslink Sweha-Reg to other population-based registries, such as The Swedish Cancer Registry, Hospital Discharge Patient Registry, National database for Acute Myocardial Infarction and Cause-of-death Register.

## Conclusion

HAE is a rare autosomal dominant disease that like other rare disorders is seldom seen by the individual physician, unless he/she is highly specialized. The potential lethality of the condition emphasizes the importance of building professional networks to develop uniform and accurate management guidelines and to reveal different outcomes including clinical, psychological and economical variables associated with the disease and its management. Establishing population-based registry of HAE is an essential first step in addressing these most important issues.

The ultimate objective of Sweha-Reg is to improve the welfare and health of patients with HAE. However, the chain from collecting the data in a registry to improved health and/or quality of life in individuals with HAE is long, complex and difficult to achieve. Therefore a more realistic intermediate objective of Sweha-Reg is to offer a fundamental source of information which can be useful for improving disease management and patient care, targeting preventive measurements to lessen co-morbidities and thereby improving quality of life in sufferers. Such an epidemiological registry is a basic prerequisite for obtaining a comprehensive and an accurate description of a rare disease such as HAE.

The information gained from Sweha-Reg can serve as a resource for quality control of treatment and patient care by clinicians and other health care providers, scientists, public health officials and researchers, by providing them a finger on the pulse of HAE in Sweden. The health care services depend on such quality controls to review their activities meaningfully.

The design of the registry permits analysis of different patient subpopulations, including pediatric patients or patients whose angioedema worsens during specific conditions such as pregnancy or hormone administration. Moreover, Sweha-Reg not only allows scientists to conduct case-control-studies but also prospective cohort studies, and to cross-link data to other population-based registries with the ability to follow up subjects to obtain information on other outcomes. Hopefully new useful patterns will emerge out of such epidemiological data.

## List of abbreviations used

C1-INH: C1 esterase inhibitor

*C1NH*: Human C1 esterase inhibitor gene

HAE: Hereditary angioedema

HAE-I: Hereditary angioedema type I

HAE-II: Hereditary angioedema type II

PIO: Primary Immunodeficiency Organization

Sweha-Reg: Swedish Hereditary Angioedema Registry

## Competing interests

The author(s) declare that they have no competing interests.

## Authors' contributions

LM has substantially contributed to conception and design and drafted the manuscript.

PN has substantially contributed to conception and design and revised the manuscript critically for important intellectual content.

JB has substantially contributed to conception and design and revised the manuscript critically for important intellectual content.

AL has substantially contributed to conception and design and revised the manuscript critically for important intellectual content.

SW has substantially contributed to conception and design and revised the manuscript critically for important intellectual content.

CFW has substantially contributed to conception and design and revised the manuscript critically for important intellectual content.

All authors read and have given final approval of the version to be published.

## Pre-publication history

The pre-publication history for this paper can be accessed here:


